# 3-Methyl-2-propionamido­butanoic acid

**DOI:** 10.1107/S1600536809007119

**Published:** 2009-03-06

**Authors:** Bohari M. Yamin, Eliyanti A. Othman

**Affiliations:** a School of Chemical Sciences and Food Technology, Universiti Kebangsaan Malaysia, UKM 43500 Bangi Selangor, Malaysia

## Abstract

The reaction of propionyl isothio­cyanate with valine was found to give the title compound, C_8_H_15_NO_3_, instead of the expected thio­urea product. The whole mol­ecule is non-planar and the carbonyl group is *cis* to the methyl­butanoic acid group across the C—N bond. Inter­molecular O—H⋯O and N—H⋯O hydrogen bonds build up a two-dimensional network developing parallel to (100).

## Related literature

For the crystal structure of *N*-propionylthio­urea, see: Yamin & Othman (2008 ▶). For bond-length data, see: Allen *et al.* (1987 ▶).
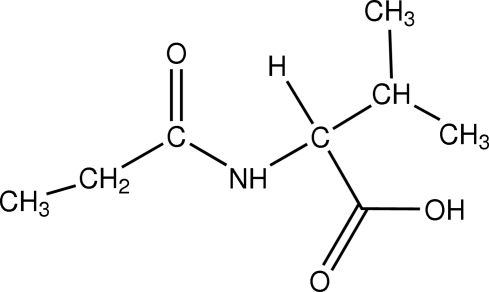


## Experimental

### 

#### Crystal data

C_8_H_15_NO_3_
*M* *_r_* = 173.21
Monoclinic, 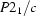

*a* = 9.477 (3) Å
*b* = 8.633 (2) Å
*c* = 12.766 (3) Å
β = 103.123 (6)°
*V* = 1017.2 (5) Å^3^
*Z* = 4
Mo *K*α radiation
μ = 0.09 mm^−1^
*T* = 298 K
0.49 × 0.33 × 0.18 mm

#### Data collection

Bruker SMART APEX CCD area-detector diffractometer
Absorption correction: multi-scan (*SADABS*; Bruker, 2000 ▶) *T*_min_ = 0.959, *T*_max_ = 0.984
5313 measured reflections
1887 independent reflections
1262 reflections with *I* > 2σ(*I*)
*R*_int_ = 0.024

#### Refinement

*R*[*F* ^2^ > 2σ(*F* ^2^)] = 0.059
*wR*(*F* ^2^) = 0.167
*S* = 1.04
1887 reflections
117 parameters
2 restraints
H atoms treated by a mixture of independent and constrained refinement
Δρ_max_ = 0.24 e Å^−3^
Δρ_min_ = −0.15 e Å^−3^

### 

Data collection: *SMART* (Bruker, 2000 ▶); cell refinement: *SAINT* (Bruker, 2000 ▶); data reduction: *SAINT*; program(s) used to solve structure: *SHELXS97* (Sheldrick, 2008 ▶); program(s) used to refine structure: *SHELXL97* (Sheldrick, 2008 ▶); molecular graphics: *ORTEPIII* (Burnett & Johnson, 1996 ▶), *ORTEP-3 for Windows* (Farrugia, 1997 ▶) and *PLATON* (Spek, 2009 ▶); software used to prepare material for publication: *SHELXL97*, *PARST* (Nardelli, 1995 ▶) and *PLATON*.

## Supplementary Material

Crystal structure: contains datablocks global, I. DOI: 10.1107/S1600536809007119/dn2429sup1.cif

Structure factors: contains datablocks I. DOI: 10.1107/S1600536809007119/dn2429Isup2.hkl

Additional supplementary materials:  crystallographic information; 3D view; checkCIF report

## Figures and Tables

**Table 1:** Hydrogen-bond geometry (Å, °)

*D*—H⋯*A*	*D*—H	H⋯*A*	*D*⋯*A*	*D*—H⋯*A*
N1—H1*D*⋯O1^i^	0.855 (18)	2.125 (18)	2.978 (3)	176.6 (16)
O2—H2*C*⋯O3^ii^	0.82 (2)	1.78 (2)	2.598 (3)	176 (2)

Symmetry codes: (i) 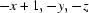; (ii) 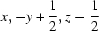.

**Fig. 1.**
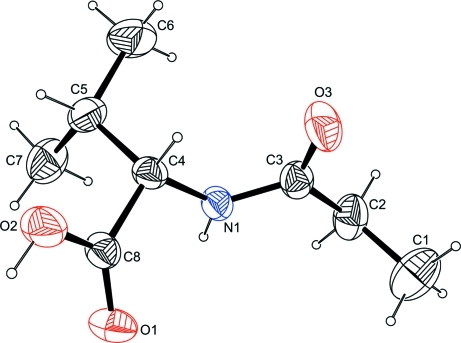
The nolecular structure of (I) with the atom-labeling scheme. Ellipsoids are drawn at the 30% probability level. H atoms are represented as small spheres of arbitrary radii. The enantiomer represented has S configuration.

**Fig. 2.**
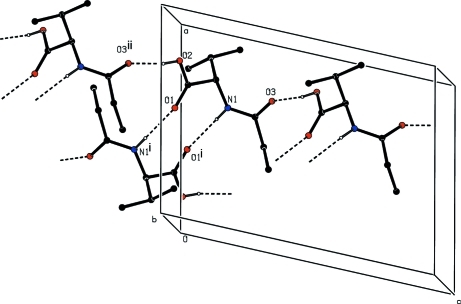
Partial packing view of I showing the H bonds network. Hydrogen bonds are shown as dashed lines. [Symmetry codes: (i) -x+1, -y, -z; (ii) x, -y+1/2, z-1/2]

## supplementary crystallographic information

### Comment

The carbonoyl isothiocyanate is a well known intermediate for the synthesis of carbonoylthiourea deriatives. However, some carbonoyl isothiocyanates such as propionyl isothiocyanate was reactive enough to give *N*-propionylthiourea (Yamin & Othman,2008) after sitrring for about 1 h. In the present study, the reaction of propionyl isothiocyanate with valine did not give the expected thiourea derivative but instead the 3-methyl-2-propionamidobutanoic acid (I), thus indicating a nucleophilic substitution of the isothiocyanato group by the amino group of the amino acid.

The molecule adopts *cis* configuration with respect to the position of the 3-methylbutanoic acid group relative to the carbonyl O3 atom across the C3—N1 bond. The bond lengths and angles are within normal ranges (Allen *et al.*, 1987). The acetamide [O3/N1/C2/C3/C4 (A)] and acetate [O1/O2/C4/C8 (B)] fragments are essentially planar with maximum deviation of 0.011 (2)Å for atom N1. The compound has a stereogenic center at C4 but the space group is centrosymmetric so the molecule exists as a racemate (R/S).

O—H···O and N—H···O intermolecular hydrogen bonds build up a two dimensional network with a corrugated iron shape developping parallel to the (1 0 0) plane.

### Experimental

A solution of propionylisothiocyanate (1.15 g, 0.01 mol) in 30 ml acetone was added into a flask containing 30 ml acetone solution of valine (1.17 g, 0.01 mol). The mixture was refluxed for 5 h. The solution was filtered and left to evaporate at room temperature. The colourless solid were obtained after one day of evaporation(yield 85%, m.p 475.1–476.3 K)

### Refinement

H atoms attached to carbon atoms were positioned geometrically and treated as riding on their parent atoms with C—H= 0.96–0.98 Å and *U*_iso_(H)= *xU*_eq_(C) where *x*=1.5 for CH_3_ group and 1.2 for CH_2_ and CH groups. The hydrogen atoms attached to nitrogen and oxygen atoms were located from Fourier difference map and refined isotropically,

### Crystal data

**Table:**

C_8_H_15_NO_3_	*F*(000) = 376
*M**_r_* = 173.21	*D*_x_ = 1.131 Mg m^−^^3^
Monoclinic, *P*2_1_/*c*	Melting point: 475.5 K
Hall symbol: -P 2ybc	Mo *K*α radiation, λ = 0.71073 Å
*a* = 9.477 (3) Å	Cell parameters from 1183 reflections
*b* = 8.633 (2) Å	θ = 2.2–25.5°
*c* = 12.766 (3) Å	µ = 0.09 mm^−^^1^
β = 103.123 (6)°	*T* = 298 K
*V* = 1017.2 (5) Å^3^	Block, colourless
*Z* = 4	0.49 × 0.33 × 0.18 mm

### Data collection

**Table:**

Bruker SMART APEX CCD area-detector diffractometer	1887 independent reflections
Radiation source: fine-focus sealed tube	1262 reflections with *I* > 2σ(*I*)
graphite	*R*_int_ = 0.024
Detector resolution: 83.66 pixels mm^-1^	θ_max_ = 25.5°, θ_min_ = 2.2°
ω scans	*h* = −10→11
Absorption correction: multi-scan (*SADABS*; Bruker, 2000)	*k* = −10→10
*T*_min_ = 0.959, *T*_max_ = 0.984	*l* = −15→8
5313 measured reflections	

### Refinement

**Table:**

Refinement on *F*^2^	Primary atom site location: structure-invariant direct methods
Least-squares matrix: full	Secondary atom site location: difference Fourier map
*R*[*F*^2^ > 2σ(*F*^2^)] = 0.059	Hydrogen site location: inferred from neighbouring sites
*wR*(*F*^2^) = 0.167	H atoms treated by a mixture of independent and constrained refinement
*S* = 1.04	*w* = 1/[σ^2^(*F*_o_^2^) + (0.0815*P*)^2^ + 0.2058*P*] where *P* = (*F*_o_^2^ + 2*F*_c_^2^)/3
1887 reflections	(Δ/σ)_max_ = 0.001
117 parameters	Δρ_max_ = 0.24 e Å^−^^3^
2 restraints	Δρ_min_ = −0.15 e Å^−^^3^

### Special details

**Table:**

Geometry. All e.s.d.'s (except the e.s.d. in the dihedral angle between two l.s. planes) are estimated using the full covariance matrix. The cell e.s.d.'s are taken into account individually in the estimation of e.s.d.'s in distances, angles and torsion angles; correlations between e.s.d.'s in cell parameters are only used when they are defined by crystal symmetry. An approximate (isotropic) treatment of cell e.s.d.'s is used for estimating e.s.d.'s involving l.s. planes.
Refinement. Refinement of *F*^2^ against ALL reflections. The weighted *R*-factor *wR* and goodness of fit *S* are based on *F*^2^, conventional *R*-factors *R* are based on *F*, with *F* set to zero for negative *F*^2^. The threshold expression of *F*^2^ > σ(*F*^2^) is used only for calculating *R*-factors(gt) *etc*. and is not relevant to the choice of reflections for refinement. *R*-factors based on *F*^2^ are statistically about twice as large as those based on *F*, and *R*- factors based on ALL data will be even larger.

### Fractional atomic coordinates and isotropic or equivalent isotropic displacement parameters (Å^2^)

**Table:**

	*x*	*y*	*z*	*U*_iso_*/*U*_eq_	
O1	0.58011 (17)	0.1690 (2)	−0.01032 (13)	0.0706 (6)	
O2	0.80400 (19)	0.2599 (2)	0.01281 (15)	0.0808 (6)	
H2C	0.775 (3)	0.309 (3)	−0.0430 (16)	0.115 (12)*	
O3	0.7243 (2)	0.0786 (2)	0.33764 (14)	0.0835 (6)	
N1	0.6507 (2)	−0.0073 (2)	0.17012 (15)	0.0523 (5)	
H1D	0.5834 (18)	−0.050 (2)	0.1230 (15)	0.058 (7)*	
C1	0.3968 (4)	0.0265 (4)	0.3214 (3)	0.1079 (12)	
H1A	0.3188	−0.0340	0.3366	0.162*	
H1B	0.3602	0.0928	0.2610	0.162*	
H1C	0.4390	0.0884	0.3830	0.162*	
C2	0.5071 (3)	−0.0769 (3)	0.2963 (2)	0.0776 (8)	
H2A	0.4623	−0.1411	0.2354	0.093*	
H2B	0.5414	−0.1449	0.3572	0.093*	
C3	0.6357 (3)	0.0049 (3)	0.27028 (19)	0.0573 (6)	
C4	0.7670 (2)	0.0664 (3)	0.13158 (16)	0.0509 (6)	
H4A	0.8199	0.1331	0.1894	0.061*	
C5	0.8765 (3)	−0.0509 (3)	0.1042 (2)	0.0684 (7)	
H5A	0.9512	0.0083	0.0797	0.082*	
C6	0.9513 (3)	−0.1423 (4)	0.2037 (3)	0.1062 (11)	
H6A	1.0190	−0.2138	0.1847	0.159*	
H6B	0.8802	−0.1983	0.2313	0.159*	
H6C	1.0020	−0.0721	0.2576	0.159*	
C7	0.8066 (3)	−0.1595 (4)	0.0136 (3)	0.0905 (9)	
H7A	0.8782	−0.2296	−0.0012	0.136*	
H7B	0.7664	−0.1002	−0.0498	0.136*	
H7C	0.7309	−0.2173	0.0345	0.136*	
C8	0.7047 (2)	0.1683 (2)	0.03739 (16)	0.0517 (6)	

### Atomic displacement parameters (Å^2^)

**Table:**

	*U*^11^	*U*^22^	*U*^33^	*U*^12^	*U*^13^	*U*^23^
O1	0.0574 (11)	0.0732 (12)	0.0697 (11)	−0.0047 (8)	−0.0100 (8)	0.0117 (8)
O2	0.0616 (11)	0.1039 (14)	0.0740 (13)	−0.0103 (10)	0.0094 (9)	0.0375 (11)
O3	0.1026 (15)	0.0943 (14)	0.0564 (11)	−0.0129 (11)	0.0237 (10)	−0.0233 (10)
N1	0.0491 (11)	0.0648 (12)	0.0422 (10)	−0.0072 (9)	0.0087 (8)	−0.0021 (9)
C1	0.088 (2)	0.088 (2)	0.162 (4)	−0.0066 (18)	0.057 (2)	0.001 (2)
C2	0.097 (2)	0.0680 (17)	0.0806 (18)	0.0026 (15)	0.0463 (16)	0.0076 (13)
C3	0.0682 (15)	0.0526 (13)	0.0538 (14)	0.0077 (12)	0.0194 (12)	−0.0015 (11)
C4	0.0442 (12)	0.0609 (13)	0.0444 (12)	−0.0073 (10)	0.0035 (9)	0.0038 (10)
C5	0.0495 (13)	0.0817 (17)	0.0755 (17)	0.0093 (12)	0.0172 (12)	0.0186 (14)
C6	0.082 (2)	0.123 (3)	0.110 (2)	0.036 (2)	0.0132 (17)	0.040 (2)
C7	0.091 (2)	0.087 (2)	0.101 (2)	0.0129 (17)	0.0370 (17)	−0.0154 (17)
C8	0.0533 (13)	0.0564 (13)	0.0438 (12)	−0.0049 (11)	0.0077 (10)	−0.0016 (10)

### Geometric parameters (Å, °)

**Table:**

O1—C8	1.200 (2)	C2—H2B	0.9700
O2—C8	1.320 (3)	C4—C8	1.499 (3)
O2—H2C	0.821 (10)	C4—C5	1.546 (3)
O3—C3	1.233 (3)	C4—H4A	0.9800
N1—C3	1.322 (3)	C5—C7	1.519 (4)
N1—C4	1.453 (3)	C5—C6	1.526 (4)
N1—H1D	0.855 (10)	C5—H5A	0.9800
C1—C2	1.464 (4)	C6—H6A	0.9600
C1—H1A	0.9600	C6—H6B	0.9600
C1—H1B	0.9600	C6—H6C	0.9600
C1—H1C	0.9600	C7—H7A	0.9600
C2—C3	1.509 (3)	C7—H7B	0.9600
C2—H2A	0.9700	C7—H7C	0.9600
			
C8—O2—H2C	114 (2)	C8—C4—H4A	107.5
C3—N1—C4	123.30 (19)	C5—C4—H4A	107.5
C3—N1—H1D	119.2 (16)	C7—C5—C6	110.8 (3)
C4—N1—H1D	117.0 (16)	C7—C5—C4	112.18 (19)
C2—C1—H1A	109.5	C6—C5—C4	111.1 (2)
C2—C1—H1B	109.5	C7—C5—H5A	107.5
H1A—C1—H1B	109.5	C6—C5—H5A	107.5
C2—C1—H1C	109.5	C4—C5—H5A	107.5
H1A—C1—H1C	109.5	C5—C6—H6A	109.5
H1B—C1—H1C	109.5	C5—C6—H6B	109.5
C1—C2—C3	114.5 (2)	H6A—C6—H6B	109.5
C1—C2—H2A	108.6	C5—C6—H6C	109.5
C3—C2—H2A	108.6	H6A—C6—H6C	109.5
C1—C2—H2B	108.6	H6B—C6—H6C	109.5
C3—C2—H2B	108.6	C5—C7—H7A	109.5
H2A—C2—H2B	107.6	C5—C7—H7B	109.5
O3—C3—N1	120.7 (2)	H7A—C7—H7B	109.5
O3—C3—C2	122.9 (2)	C5—C7—H7C	109.5
N1—C3—C2	116.4 (2)	H7A—C7—H7C	109.5
N1—C4—C8	109.76 (17)	H7B—C7—H7C	109.5
N1—C4—C5	112.93 (19)	O1—C8—O2	123.3 (2)
C8—C4—C5	111.44 (18)	O1—C8—C4	125.0 (2)
N1—C4—H4A	107.5	O2—C8—C4	111.72 (19)
			
C4—N1—C3—O3	−1.7 (3)	C8—C4—C5—C7	−61.5 (3)
C4—N1—C3—C2	179.0 (2)	N1—C4—C5—C6	−62.0 (3)
C1—C2—C3—O3	68.0 (4)	C8—C4—C5—C6	173.9 (2)
C1—C2—C3—N1	−112.7 (3)	N1—C4—C8—O1	−11.6 (3)
C3—N1—C4—C8	−123.5 (2)	C5—C4—C8—O1	114.2 (3)
C3—N1—C4—C5	111.5 (2)	N1—C4—C8—O2	167.87 (19)
N1—C4—C5—C7	62.6 (3)	C5—C4—C8—O2	−66.3 (2)

### Hydrogen-bond geometry (Å, °)

**Table:**

*D*—H···*A*	*D*—H	H···*A*	*D*···*A*	*D*—H···*A*
N1—H1D···O1^i^	0.86 (2)	2.13 (2)	2.978 (3)	177 (2)
O2—H2C···O3^ii^	0.82 (2)	1.78 (2)	2.598 (3)	176 (2)

Symmetry codes: (i) −*x*+1, −*y*, −*z*; (ii) *x*, −*y*+1/2, *z*−1/2.
